# The First Record of the Slaty‐Breasted Rail *Lewinia striata* Inhabiting the Invasive *Spartina alterniflora* in Dafeng, Yancheng, China

**DOI:** 10.1002/ece3.71204

**Published:** 2025-04-02

**Authors:** Zhen‐Qi Wang, Da‐Wei Wu, Wei Hu, Chang‐Hu Lu

**Affiliations:** ^1^ Nanjing Forestry University Nanjing China

**Keywords:** biological invasion, habitat use, Slaty‐breasted rail, smooth cordgrass

## Abstract

In the context of the prolonged invasion of smooth cordgrass *Spartina alterniflora* , numerous native birds have progressively adapted to this invasive plant. Not only do certain non‐specialist birds demonstrate an ability to inhabit and utilize smooth cordgrass, but a subset of specialist birds is also detected in the habitat as well. In this study, we provide evidence of the Slaty‐breasted Rail *Lewinia striata* (Rallidae) occurring in smooth cordgrass, which had not been previously documented. These rails exhibit a significant reliance on the invaded vegetative community during their breeding season, engaging in foraging, vocalization, and even successful reproduction within the smooth cordgrass. The observations of Slaty‐breasted Rail utilizing invasive smooth cordgrass highlight the possibility that, as natural wetlands continue to decline, birds that are highly dependent on marsh ecosystems may increasingly resort to the invasive *Spartina* community as a critical refuge. Such a transition is poised to impact their distribution, foraging, and reproduction. With the backdrop of large‐scale removal of smooth cordgrass on the China coast, the disappearance of smooth cordgrass may harm the population of these birds. Further dedicated investigations and tracking of more individuals are needed to understand the specific ecological impact on Slaty‐breasted Rail.

## Abstract 2

In this study, we investigate a previously undocumented Rallidae species, the slaty‐breasted rail Lewinia striata, living in smooth cordgrass. These rails exhibit a significant reliance on the invaded vegetative community during their breeding season, engaging in foraging, vocalization, and even successful reproduction within the smooth cordgrass.

## 1 Introduction

As globalization progresses, plant invasions caused by deliberate or unintentional introductions continue to increase worldwide (Gioria et al. 2023). Invasive plants are usually defined as non‐native species that, when introduced to a new environment, have the potential to spread rapidly and cause harm to the ecosystem through various ways and threaten local biodiversity and habitat quality (Kumar and Prasad 2014). Numerous native species are driven toward extinction because of invasive plants (Bellard et al. 2016; Stewart et al. 2021). Therefore, plant invasion is considered one of the most common causes of biodiversity loss (Simberloff 2007). However, the impacts of invasive plants are not entirely negative, and the simplistic dichotomy of categorizing native plants as beneficial and invasive plants as detrimental has been challenged (Goodenough 2010). Invasive species can also benefit native species through mechanisms such as habitat modification, competitive release, and predator release (Rodriguez 2006; Overton et al. 2014). To comprehend impacts and make decisions regarding conservation actions, it is crucial to understand and study how native species respond to invasive alien species (Humair et al. 2014; Pyšek et al. 2020).

*Spartina alterniflora* , commonly known as smooth cordgrass, is native to the eastern coast of North America and it was introduced by multiple countries in the last century as a flood control and soil erosion prevention plant for coastal areas (Ranwell and Downing 1960; Daehler and Strong 1996). Due to its exceptionally strong spreading capability and extensive coverage, it has now become one of the most globally notorious invasive plants (Ren et al. 2021). Because of its effective roles in facilitating silt deposition, land reclamation, and shoreline protection against waves, China introduced smooth cordgrass in the 1970s (Deng et al. 2006; Chung 2006). Over the subsequent 40 years, smooth cordgrass has continuously expanded and now covers most of the coastline of China (Mao et al. 2019). Smooth cordgrass is leading to a significant reduction in coastal mudflat areas and causing severe impacts on local ecosystems and economic activities (Zhang et al. 2017). In 2003, smooth cordgrass was listed as one of the top 16 alien species in China (State Environmental Protection Administration & Chinese Academy of Sciences, 2003). The Chinese government mandated its full eradication from coastal areas. In 2023, large‐scale eradication efforts against smooth cordgrass commenced in coastal regions of China, with the goal of complete eradication by 2025.

Studies have indicated that the invasion of smooth cordgrass in coastal regions of China occupies bare beaches and causes a decline in local biodiversity due to habitat modification (Zuo et al. 2012; Yu et al. 2022). Among the effects, the negative impact on shorebirds is particularly severe, as, with the drastic reduction in bare beach, there has been a significant decrease in the number of available foraging and roosting sites for them (Gan et al. 2009; Jackson et al. 2021). However, some native passerine birds have properly utilized invaded habitats such as Marsh grassbird (*Helopsaltes pryeri*) (Ma et al. 2014). They inhabit smooth cordgrass areas and depend on exotic plants for their needs, even for reproduction (Chen et al. 2023). This change is likely correlated with the duration of the smooth cordgrass invasion (Chen et al. 2022). Additionally, according to previous research and reports, native birds utilizing invasive smooth cordgrass are predominantly nonspecialised passerine birds, which are commonly found across various habitats and exhibit great adaptability (Ma et al. 2014; Chen et al. 2022). Recently, there have also been reports indicating that specialized birds such as Reed Parrotbills (*Calamornis heudei*) can also utilize smooth cordgrass (Dawei et al. 2023). Due to human disturbances and the ongoing reduction of natural wetlands, more and more native birds are attempting to utilize invasive vegetation, although the invaded habitats may be an ecological trap for them (Ma et al. 2014; Chen et al. 2023).

The Slaty‐breasted Rail (*Lewinia striata*) is a waterbird of the family Rallidae and is a widely distributed species in southern China and southeastern Asia (BirdLife International 2024). The Slaty‐breasted Rail can utilize various types of wetlands such as marshes, bogs, mangroves, swamps, wet grasslands, and paddy fields, and prefers denser vegetation such as the mangrove *Kandelia candel* and usually forages in the morning and evening (BirdLife International 2024). The species is listed as Least Concern under the IUCN Red List of Threatened Species (BirdLife International 2024); however, in some areas they are listed as locally vulnerable because of their small population, loss and degradation of habitat, illegal hunting, and disturbance (Inskipp et al. 2016). The global population size and overall population trend of this species are both unknown (BirdLife International 2024). Nationally in China, they are still extremely rare to discover in the wild. At present, the ecological research on the Slaty‐breasted Rail is limited. In this study, we report the first known observation of the Slaty‐breasted Rail utilizing wetlands dominated by the invasive *Spartina alterniflora* . This finding could help to expand our understanding of ecological plasticity and habitat utilization of this species, thereby providing insights for assessing the impact of invasive plants on native birds.

## 2 Materials and Methods

**FIGURE 1:**
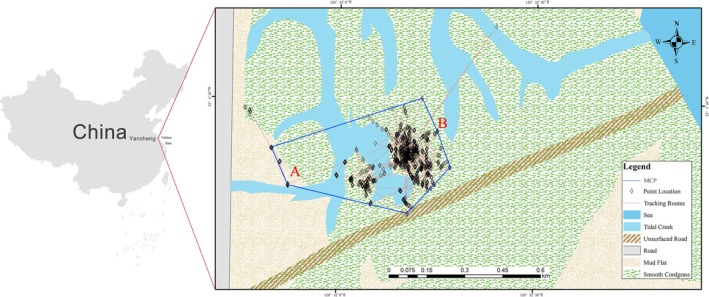
The movement and home range of the tracking slaty‐breasted rail from July 13th to August 1st, 2023.

The study site is in a *Spartina* salt marsh (E120.86830231, N33.07108837), which is at the edge of the third core area of Dafeng Milu National Nature Reserve, a Ramsar wetland in Yancheng city, Jiangsu province, China. The area covers approximately 1.7 km^2^, with a perimeter of approximately 5.6 km (see Figure 1). The nearby area of the study site is surrounded by artificial fish pond farming zones, wheat fields, and scrub forests. The tidal creeks leading to the sea are also occupied by the invasive *Spartina alterniflora* .

Our surveys did not include the use of audio playback, a common method used in marsh bird studies (Conway 2011). Instead, we incorporated trap checks as part of the survey process, ensuring that all detected rails were observed and documented. From May to September 2023, we conducted monthly surveys of the Slaty‐breasted Rail in the area, each one lasting at least 2 h. We utilized door‐closing iron cages as traps, baited with sausages and small crabs to attract the rails. Those traps were placed along pre‐determined transects identified through prior field assessments. Navigating salt marsh environments can be challenging; therefore, transect routes were carefully selected to maximize accessibility and facilitate efficient survey execution. Traps were spaced approximately 20 m apart, with a total of 30 traps deployed along the selected route. During surveys, we checked the traps along the transect and recorded any observed rails using a Nikon Z9 camera with a 600 mm lens. For those not visually sighted but heard vocalizing, we recorded their calls using an iPhone 13. Additionally, we fitted the captured adult Slaty‐breasted Rail with a 4.86 g Global Positioning System‐Global System for Mobile Communication transmitter (HQBG1204, Hunan Global Messenger Technology Co. Ltd. Hunan, China). The tracking device worked by positioning frequency once every 4 h and transmitting data for every five positions collected. The transmitter was attached to the back of the Slaty‐breasted Rail using the leg‐loop harness. We confirmed that the total weight of the device was not more than 3% of the rail's body weight.

A: the site where we caught and released the Slaty‐breasted Rail; B: the last location point before the tracking signal was lost. Source of the background image: Using the Copernicus Browser (https://browser.dataspace.copernicus.eu), we downloaded 2022 Sentinel‐2 remote sensing images. The imagery was processed and clipped to the experimental site range via ENVI 5.6, and supervised classification was performed combination with field surveys.

## 3 Results

**FIGURE 2:**
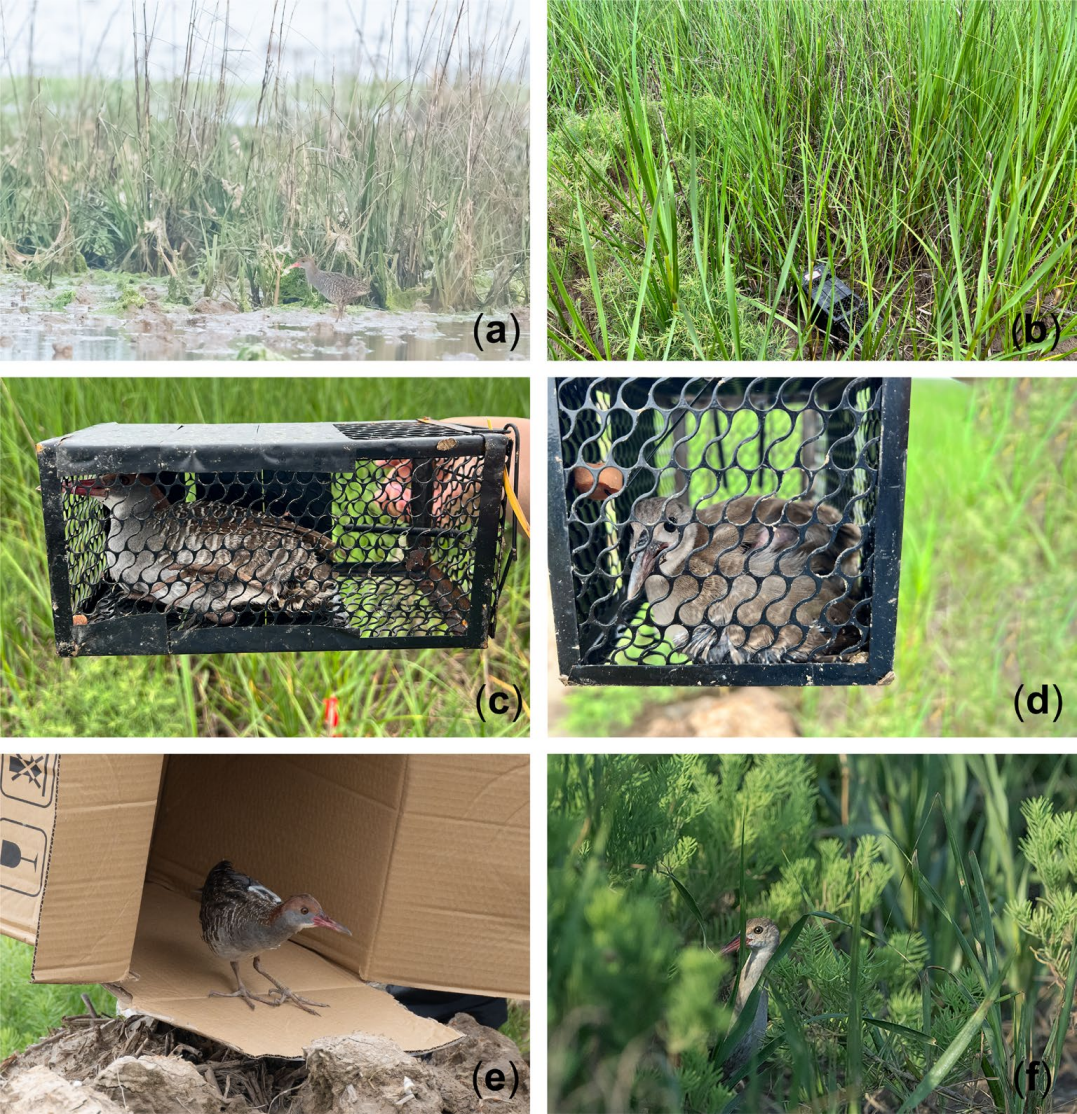
(a) The first photographic record of the slaty‐breasted rail in June; (b) habitat by trap; (c) the first capture of an adult rail in July; (d) the first capture of a fledgling rail in July; (e) the slaty‐breasted rail release; (f) the first photographic record of a sub‐adult in August.

After five surveys of Slaty‐breasted Rails in the smooth cordgrass salt marsh, we detected activity of the rail four times in this habitat. The first observation of Slaty‐breasted Rail utilizing the smooth cordgrass habitat occurred on the afternoon of May 15th, 2023. While conducting the survey in June, we obtained the first clear picture (see Figure 2) of Slaty‐breasted Rail foraging among the smooth cordgrass. Upon sighting us, that individual promptly retreated into dense vegetation for cover. Subsequently, we heard its call and also recorded its vocalizations. On July 11, we captured 2 Slaty‐breasted Rails, one adult and one fledgling. The adult rail (weight 162.8 g) was released after being fitted with a GPS transmitter (HQBG1204), showing no abnormal behavior. We were unable to attach a transmitter to the fledgling due to its weight and age, so it was released the same day. After 20 days of tracking, the device indicated abnormal activity on August 2, 2023, possibly due to device detachment or death of the rail. During this period, we did not observe the individual wearing the device. The final location was pinpointed deep within the smooth cordgrass salt marsh (see Figure 1). Based on the last location provided by the tracking device, we attempted to retrieve it, but unfortunately, we failed to find it. The tracking data revealed that the Slaty‐breasted Rail remained exclusively within the smooth cordgrass salt marsh for the entire 20 days after its release, with its activities range encompassing the initial sighting location (see Figure 1). Based on GPS tracking data, the home range was estimated at 269,941.23 m^2^ using the 99% Minimum Convex Polygon (MCP) method, which removed 1% of the most extreme locations to provide a more stable estimation of space use. On August 8th, we recorded the first image of a subadult Slaty‐breasted Rail (see Figure 2) foraging under the invasive smooth cordgrass, which also marked the last observation of the rail in the invaded habitat. From May to September 2023, we totally conducted five surveys, during which we observed Slaty‐breasted Rails active in the smooth cordgrass on all occasions except for the last time in September (see Table 1).

**TABLE 1:** Detection time and results.

Date	Time	Detecting method	Number	Age
2023.05.15	18:09	Sight, hearing	1 Individual	Adult
2023.06.09	19:01	Photograph, hearing	1 Individual	Adult
2023.07.11	11:11, 11:52	Capture	2 Individuals	Adult, fledgling
2023.08.08	16:56	Sight	1 Individual	Sub‐adult
2023.09	—	—	—	—

*Note:* “Number” means the number of individual rails were observed.

## 4 Discussion

The observations of Slaty‐breasted Rails utilizing smooth cordgrass as a habitat are not random occurrences. As the duration of invasion increases, more and more native birds are entering these invaded habitats. Some species, especially those with high adaptability, may utilize these new habitats for breeding and foraging (Catling 2005; Blackburn et al. 2011; Schirmel et al. 2016). The degree to which birds adapt to these invaded habitats varies. Species with flexible behaviors and broad dietary preferences can adapt more quickly and effectively to new habitats (Dylewski et al. 2019). For example, in South America, species like the Merida Wren ( *Cistothorus meridae* ) and Song Sparrow ( *Melospiza melodia* ) have started breeding in invaded smooth cordgrass habitats (Nordby et al. 2009; Lampert et al. 2014). In China, the non‐native Marsh Grassbird (*Helopsaltes pryeri*) has entered *Spartina* habitats in the Dongtan Wetland on Chongming Island, Shanghai, and bred successfully (Ma et al. 2014). A similar pattern has also been observed in Yancheng, Jiangsu Province, where native species such as Plain Prinia ( *Prinia inornata* ) and Vinous‐throated Parrotbill ( *Sinosuthora webbiana* ) have utilized the smooth cordgrass effectively and bred successfully in smooth cordgrass habitats (Chen et al. 2022). Initially, the presence of smooth cordgrass may attract native species due to reduced competition and dense vegetation (Nordby et al. 2009; Sun et al. 2020). However, factors such as diminished food resources, tidal inundation, and heightened predation risk ultimately render smooth cordgrass a potential ecological trap (Chen et al. 2023). While some native birds enter smooth cordgrass for breeding, research indicates that their nests are more susceptible to destruction by small mammals, leading to a decrease in reproductive success rates (Nordby et al. 2009; Fisher and Davis 2011). This outcome may be due to the absence of higher‐level predators in the invasive vegetative community.

The Slaty‐breasted Rail is highly dependent on wetland habitats and is known for its secretive behavior. This species prefers habitats with dense vegetation (Mace and Glory 2016), and smooth cordgrass provides the vegetation density commonly used by Slaty‐breasted Rails. Throughout the summer, we observed Slaty‐breasted Rails inhabiting the smooth cordgrass, with evidence suggesting that they might have successfully bred within the habitat. This is also the first time the Slaty‐breasted Rail has been documented in the Yancheng region. The study site was once predominantly bare beaches, and the surrounding area consists of artificial fish ponds, wheat fields, and scrub forests, which were not suitable for rails' habitat. Over the years, the invasive smooth cordgrass occupied the tidal flats. The invasion of cordgrass has significantly altered the habitat, with a monoculture of living and dead cordgrass vegetation covering the entire region. This dense vegetation also provides potential habitat for birds, supporting behaviors such as nesting and reproduction to some extent. Although we have not yet discovered any nesting sites of Slaty‐breasted Rail in the smooth cordgrass, the observation of five individuals, as well as the tracking of an adult in the area, suggests that Slaty‐breasted Rails are effectively utilizing the smooth cordgrass. We hypothesize that the invasive *Spartina* community may have become an important habitat for these rails. However, it can't be ruled out that the reduction of natural wetlands in the surrounding landscape may have forced the Slaty‐breasted Rails to enter the invaded habitat. The removal of *Spartina alterniflora* could potentially lead to the local disappearance of the Slaty‐breasted Rail. Therefore, further research and additional samples are needed to test this hypothesis. Habitat quality, including factors such as food availability, humidity, and temperature, should also be assessed for further insight.

Currently, the Chinese government is vigorously promoting the removal of smooth cordgrass in coastal areas to prevent its expansion and to restore habitats for both fisheries production and waders. Preliminary results from regions where smooth cordgrass has been removed show significant increases in populations of wading birds (Lyu et al. 2023). The study area is a key habitat for various rare waterbirds, such as Spoon‐billed Sandpiper (*Calidris pygmaea*) and Nordmann's Greenshank ( *Tringa guttifer* ). Removing *Spartina alterniflora* to restore mudflats is beneficial for the habitat of these waterbirds. However, the removal of smooth cordgrass is solely aimed at restoring bare tidal flats, without the restoration of any native vegetation. One of the key questions for future research will be to assess the impact of this project on native bird species that have developed a dependence on the invaded habitats due to the long‐term presence of smooth cordgrass. On the west coast of the United States, the history of invasive smooth cordgrass and its hybridization with native cordgrass is even more extensive. For instance, the Ridgway's Rail (*Rallus obsoletus*) has become increasingly reliant on the introduced *Spartina* species, having adapted to these habitats (Lampert et al. 2014). However, the ongoing eradication of *Spartina alterniflora* poses a significant threat to these endangered rails, as their survival is increasingly tied to the presence of this invasive vegetation and hybrid species it produced (Casazza et al. 2016). Similarly, in South America, the Mangrove Rail ( *Rallus longirostris* ) has utilized the exotic tanner grass ( *Urochloa arrecta* ) in salt marshes as a key habitat, and the removal of these invasive plants has led to the disappearance of the Mangrove Rail (Bornschein et al. 2022). Nowadays, a similar process is quietly unfolding in China, and the Slaty‐breasted Rail may also be facing this survival challenge. If more natural wetlands are not restored, the removal of *Spartina alterniflora* is likely to have a more detrimental impact on this species. Therefore, retaining a small portion of *Spartina* habitat while controlling its expansion is currently the most direct and feasible management approach in this region.

## Author Contributions

**Zhen‐Qi Wang:** conceptualization (lead), data curation (lead), investigation (lead), methodology (lead), software (equal), visualization (equal), writing – original draft (lead). **Da‐Wei Wu:** data curation (equal), investigation (equal), software (equal), visualization (equal). **Wei Hu:** investigation (equal). **Chang‐Hu Lu:** project administration (equal), resources (equal), supervision (equal), writing – review and editing (equal).

## Ethics Statement

The study was conducted according to the guidelines of the Declaration of Helsinki and approved by the Animal Ethics committee of Nanjing Forestry University (2024046).

## Conflicts of Interest

The authors declare no conflicts of interest.

## Data Availability

The authors have nothing to report.
